# Ambient air pollution and non-communicable respiratory illness in sub-Saharan Africa: a systematic review of the literature

**DOI:** 10.1186/s12940-022-00852-0

**Published:** 2022-04-14

**Authors:** Bailey E. Glenn, Leon M. Espira, Miles C. Larson, Peter S. Larson

**Affiliations:** 1 Department of Biostatistics and Epidemiology, School of Public Health and Health Sciences, University of Massachusetts Amherst, Amherst, MA USA; 2 Center for Global Health Equity, University of Michigan, Ann Arbor, USA; 3 Washtenaw Community College, Ann Arbor, MI USA; 4 Social Environment and Health Program, Survey Research Center, Institute for Social Research, University of Michigan, Ann Arbor, MI USA; 5 Department of Epidemiology, School of Public Health, University of Michigan, Ann Arbor, MI USA

**Keywords:** Respiratory, Air pollution, Noncommunicable respiratory disease, Asthma, Bronchitis, Allergic rhinitis, Chronic obstructive pulmonary disease, Particulate matter, Air quality, Environmental epidemiology, Global health

## Abstract

**Introduction:**

Aerosol pollutants are known to raise the risk of development of non-communicable respiratory diseases (NCRDs) such as asthma, chronic bronchitis, chronic obstructive pulmonary disease, and allergic rhinitis. Sub-Saharan Africa’s rapid pace of urbanization, economic expansion, and population growth raise concerns of increasing incidence of NCRDs. This research characterizes the state of research on pollution and NCRDs in the 46 countries of Sub-Saharan Africa (SSA). This research systematically reviewed the literature on studies of asthma; chronic bronchitis; allergic rhinitis; and air pollutants such as particulate matter, ozone, NOx, and sulfuric oxide.

**Methods:**

We searched three major databases (PubMed, Web of Science, and Scopus) using the key words “asthma”, “chronic bronchitis”, “allergic rhinitis”, and “COPD” with “carbon monoxide (CO)”, “sulfuric oxide (SO)”, “ozone (O3)”, “nitrogen dioxide (NO2)”, and “particulate matter (PM)”, restricting the search to the 46 countries that comprise SSA. Only papers published in scholarly journals with a defined health outcome in individuals and which tested associations with explicitly measured or modelled air exposures were considered for inclusion. All candidate papers were entered into a database for review.

**Results:**

We found a total of 362 unique research papers in the initial search of the three databases. Among these, 14 met the inclusion criteria. These papers comprised studies from just five countries. Nine papers were from South Africa; two from Malawi; and one each from Ghana, Namibia, and Nigeria. Most studies were cross-sectional. Exposures to ambient air pollutants were measured using spectrometry and chromatography. Some studies created composite measures of air pollution using a range of data layers. NCRD outcomes were measured by self-reported health status and measures of lung function (spirometry). Populations of interest were primarily schoolchildren, though a few studies focused on secondary school students and adults.

**Conclusions:**

The paucity of research on NCRDs and ambient air pollutant exposures is pronounced within the African continent. While capacity to measure air quality in SSA is high, studies targeting NCRDs should work to draw attention to questions of outdoor air pollution and health. As the climate changes and SSA economies expand and countries urbanize, these questions will become increasingly important.

## Introduction

Sub-Saharan Africa (SSA), comprised of 46 countries according to the United Nations Development Programme [1], is experiencing unprecedented levels of economic and population growth. The share of SSA’s population living in urban areas has increased from 15% in 1960 to more than 40% in 2020 [2]. Africa’s urban population is expected to climb to more than 1.3 billion people by 2050, and cities such as Kinshasa in the Democratic Republic of Congo and Lago in Nigeria will be among the largest cities in the world [3]. Urbanization, combined with climate change, will be among the greatest challenges to SSA’s development and to the world [4].

SSA is also facing the challenge of adapting public health programs and resources to meet the changing health needs of an increasing population. While average life expectancy in all SSA countries has increased by more than 20 years since 1960 [5], the incidence of health threats from chronic conditions is growing [6]. New cases of non-communicable diseases (NCDs) such as cancer [7, 8], heart disease, hypertension [9, 10], and diabetes [11, 12] are becoming more and more numerous, even among less affluent populations [6, 13, 14]. Treatment options for poor people suffering from non-communicable and chronic health problems are often limited due to their high cost to fragile health systems [15] and, even when available, are catastrophically unaffordable for most patients [16, 17].

Incidence of respiratory conditions and respiratory related mortality are also expected to increase with urbanization and climate change. Rapid urbanization increases exposures to aerosol pollutants such as carbon monoxide (CO), sulfuric oxide (SO), ozone (O3), nitrogen dioxide (NO2), and particulate matter (PM)[18]. In high-income countries, exposures to these pollutants have been associated with increased risks for a number of chronic respiratory problems, including chronic bronchitis, asthma, and allergic rhinitis. For example, PM exposures have been associated with the development of conditions leading to larger numbers of hospitalizations in several contexts [19]. Long-term exposures to PM have been associated with the incidence of chronic bronchitis [20], childhood asthma [21–23], and allergic rhinitis [24]. NO2 exposures have been found to exacerbate pre-existing respiratory health problems [25]. Cohort studies in Europe have shown that long-term exposures to NO and PMx are associated with rhinitis [26].

A recent review of research from 27 countries in Africa indicated that air pollutants such as PM, CO, NO2, and SO2 commonly exceed WHO guidelines [27]. Indoor pollution exposures, primarily through tobacco or the use of biomass cooking fuels, have been shown to have impacts on public health in low-income countries [28, 29]. In SSA countries, research has shown that indoor cooking using biomass fuels is associated with a number of non-communicable respiratory diseases (NCRDs), including acute respiratory infections [30], tuberculosis [31], chronic obstructive pulmonary disease (COPD) [32], and asthma [33]. Less well understood, however, are the associations between ambient (outdoor) air exposures and NCRDs in SSA. As SSA countries develop and urbanize, questions regarding air quality and public health in the form of NCRDs will become increasingly salient.

This research characterizes the state of research in SSA countries on NCRDs commonly associated with exposures to air pollutants by conducting a systematic review of the literature. This work builds on previously published reviews such as that by Coker and Kizito (2018) by examining associations between specific outcome and exposure variables as well as providing a more detailed review of current literature than has been done previously [34]. We particularly focus on research studies that specifically document the public health impacts of ambient pollution exposures on individual health through empirical methods.

## Methods

### Search strategy and selection criteria

We conducted a systematic review of the databases PubMed, Web of Science, and Scopus to locate scholarly research literature published up to August 21, 2021.

For this review, we searched all three databases for abstracts that contained the keywords “asthma”, “chronic bronchitis”, “allergic rhinitis”, “COPD”, or “chronic obstructive pulmonary disease”; along with the exposure terms “carbon monoxide”, “CO”, “sulfur”, “SO”, “ozone”, “O3”, “nitrogen”, “NO2”, “NOx”, “particulate matter”, “PM”, “PM10”, and “PM2.5”. We restricted the search to the 46 countries considered to comprise SSA [1]. See Table 1 for full search terms and search strategies.

**Table 1:** Search terms and search strategy used to identify studies on measured air pollution exposures and respiratory disease

Search field	PubMed	Web of science	Scopus
1	(Asthma OR “chronic bronchitis” OR “allergic rhinitis” OR “COPD” OR “chronic obstructive pulmonary disease”)	(AB=Asthma OR AB=“chronic bronchitis” OR AB=“allergic rhinitis” OR AB=“COPD” OR AB=“chronic obstructive pulmonary disease”)	ABS(Asthma OR “chronic bronchitis” OR “allergic rhinitis” OR “COPD” OR “chronic obstructive pulmonary disease”)
2	(“air pollution” OR “climate change” OR “urbanization” OR “Sulfur” OR “SO” OR “Nitrogen” OR “NOx” OR “ozone” OR “carbon monoxide” OR “PM2.5” OR “PM10” OR “particulate matter”)	(AB=“air pollution” OR AB=“climate change” OR AB=“urbanization” OR AB=“Sulfur” OR AB=“SO” OR AB=“Nitrogen” OR AB=“NOx” OR AB=“ozone” OR AB=“carbon monoxide” OR AB=“PM2.5” OR AB=“PM10” OR AB=“particulate matter”)	ABS(“air pollution” OR “climate change” OR “urbanization” OR “Sulfur” OR “SO” OR “Nitrogen” OR “NOx” OR “ozone” OR “carbon monoxide” OR “PM2.5” OR “PM10” OR “particulate matter”)
3	(“Africa” OR “Angola” OR “Benin” OR “Botswana” OR “Burkina Faso” OR “Burundi” OR “Cameroon” OR “Central African Republic” OR “Chad” OR “Congo” OR “Cote d’Ivoire” OR “Eritrea” OR “Ethiopia” OR “Gabon” OR “Gambia” OR “Ghana” OR “Guinea” OR “Guinea-Bissau” OR “Kenya” OR “Lesotho” OR “Liberia” OR “Madagascar” OR “Malawi” OR “Mali” OR “Mauritania” OR “Mauritius” OR “Mozambique” OR “Namibia” OR “Niger” OR “Nigeria” OR “Rwanda” OR “Senegal” OR “Sierra Leone” OR “Somalia” OR “Tanzania” OR “Togo” OR “Uganda” OR “Zaire” OR “Zambia” OR “Zimbabwe” OR “South Africa”)	(AB=“Africa” OR AB=“Angola” OR AB=“Benin” OR AB=“Botswana” OR AB=“Burkina Faso” OR AB=“Burundi” OR AB=“Cameroon” OR AB=“Central African Republic” OR AB=“Chad” OR AB=“Congo” OR AB=“Cote d’Ivoire” OR AB=“Eritrea” OR AB=“Ethiopia” OR AB=“Gabon” OR AB=“Gambia” OR AB=“Ghana” OR AB=“Guinea” OR AB=“Guinea-Bissau” OR AB=“Kenya” OR AB=“Lesotho” OR AB=“Liberia” OR AB=“Madagascar” OR AB=“Malawi” OR AB=“Mali” OR AB=“Mauritania” OR AB=“Mauritius” OR AB=“Mozambique” OR AB=“Namibia” OR AB=“Niger” OR AB=“Nigeria” OR AB=“Rwanda” OR AB=“Senegal” OR AB=“Sierra Leone” OR AB=“Somalia” OR AB=“Tanzania” OR AB=“Togo” OR AB=“Uganda” OR AB=“Zaire” OR AB=“Zambia” OR AB=“Zimbabwe” OR AB=“south africa”)	ABS(“Africa” OR “Angola” OR “Benin” OR “Botswana” OR “Burkina Faso” OR “Burundi” OR “Cameroon” OR “Central African Republic” OR “Chad” OR “Congo” OR “Cote d’Ivoire” OR “Eritrea” OR “Ethiopia” OR “Gabon” OR “Gambia” OR “Ghana” OR “Guinea” OR “Guinea-Bissau” OR “Kenya” OR “Lesotho” OR “Liberia” OR “Madagascar” OR “Malawi” OR “Mali” OR “Mauritania” OR “Mauritius” OR “Mozambique” OR “Namibia” OR “Niger” OR “Nigeria” OR “Rwanda” OR “Senegal” OR “Sierra Leone” OR “Somalia” OR “Tanzania” OR “Togo” OR “Uganda” OR “Zaire” OR “Zambia” OR “Zimbabwe” OR “south africa”)

### Inclusion/Exclusion criteria

All candidate papers from the initial search results were entered into a database for review. Duplicate publications were removed from the database. We removed conference proceedings, review papers, and papers where the full text was not available. We excluded papers that did not include human subjects, such as animal studies, pharmaceutical development, basic scientific (lab-based) studies, or studies on environmental sampling. We then removed papers that did not sample individuals within Sub-Saharan Africa. For the remaining papers, we thoroughly examined each to find publications that tested a relationship between a defined human health outcome pertaining to asthma, chronic bronchitis, or allergic rhinitis; and one or more specific and mechanically measured ambient or environmental exposures. We included only papers that conducted observational studies and tested associations of exposures and outcomes at the individual level. Household-level studies and studies of disease counts in aggregate were excluded.

Papers on indoor (i.e. cooking fuels) exposures, non specific air pollutant exposures (e.g. “traffic” or “car exhaust”), and occupational exposures that exclusively focused on workers were excluded, as we sought to test associations between ambient (outdoor) exposures to aerosol pollutants and health outcomes. Only papers written in English and published in scholarly journals were included in the analysis. Each paper included in the database was analyzed further by all authors to ensure each paper met the inclusion and exclusion criteria.

### Data extraction

For the papers that met the full inclusion criteria listed above, we obtained full texts and extracted relevant variables of interest. When available, we extracted the following variables: disease measured, study type, study location, and exposure. We created a master database of all papers reviewed. Team members then independently reviewed each entry for accuracy.

### Data definition

We classified health outcomes into four groups: asthma, chronic bronchitis, allergic rhinitis, COPD, or a combination of two or more. Study types were grouped into cross-sectional, cohort, case-control, ecological, and intervention study categories.

For the purposes of this systematic review, we included papers that had a defined NCRD outcome (asthma, chronic bronchitis, COPD, or allergic rhinitis), restricting the definition to any of the following: a self-reported case status, a physician-diagnosed case, or an author-diagnosed case of disease via an assessment tool.

### Quality and bias assessment

We used the Appraisal tool for Cross-Sectional Studies (AXIS tool) to assess research quality and internal validity of each relevant study [35]. Case-control and cohort studies were evaluated using the eight-item Newcastle-Ottawa Scale [36]. Cross-sectional studies were scored on a 20-point scale, while case-control and cohort studies were scored on a 10-point scale, based on the respective assessment tools used.

## Results

**Fig. 1:**
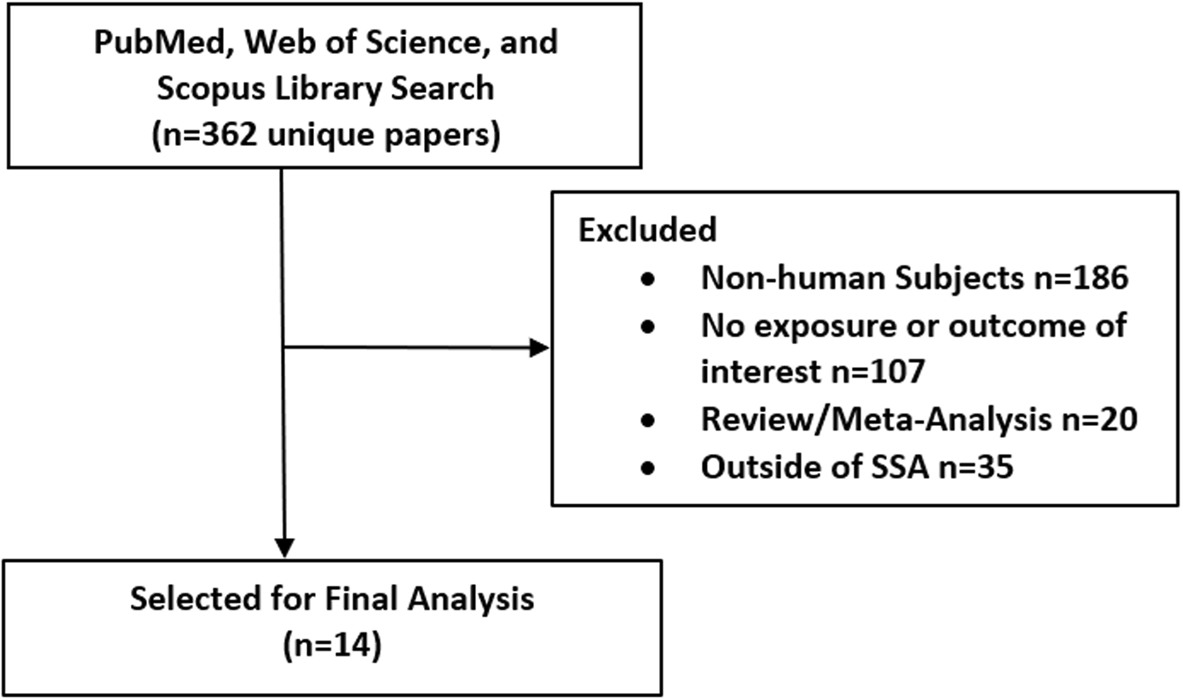
Description of the primary literature search process

Using the search criteria, we collected 249 articles on PubMed, 141 articles on Web of Science and 190 articles on Scopus. There were 362 unique papers after removing duplicates. Of these, 186 were on non-human subjects. These included 67 papers on animal studies (e.g. guinea pigs, rats), and 119 papers on lab/pharma/bio studies, and policy. We found 35 papers that were in regions outside SSA, 20 review/meta-analysis papers, and 107 papers of human and observational studies that did not have an exposure or outcome of interest. After excluding all of those, 14 remained. See Fig. 1 for a visualization of the search process.

**Fig. 2:**
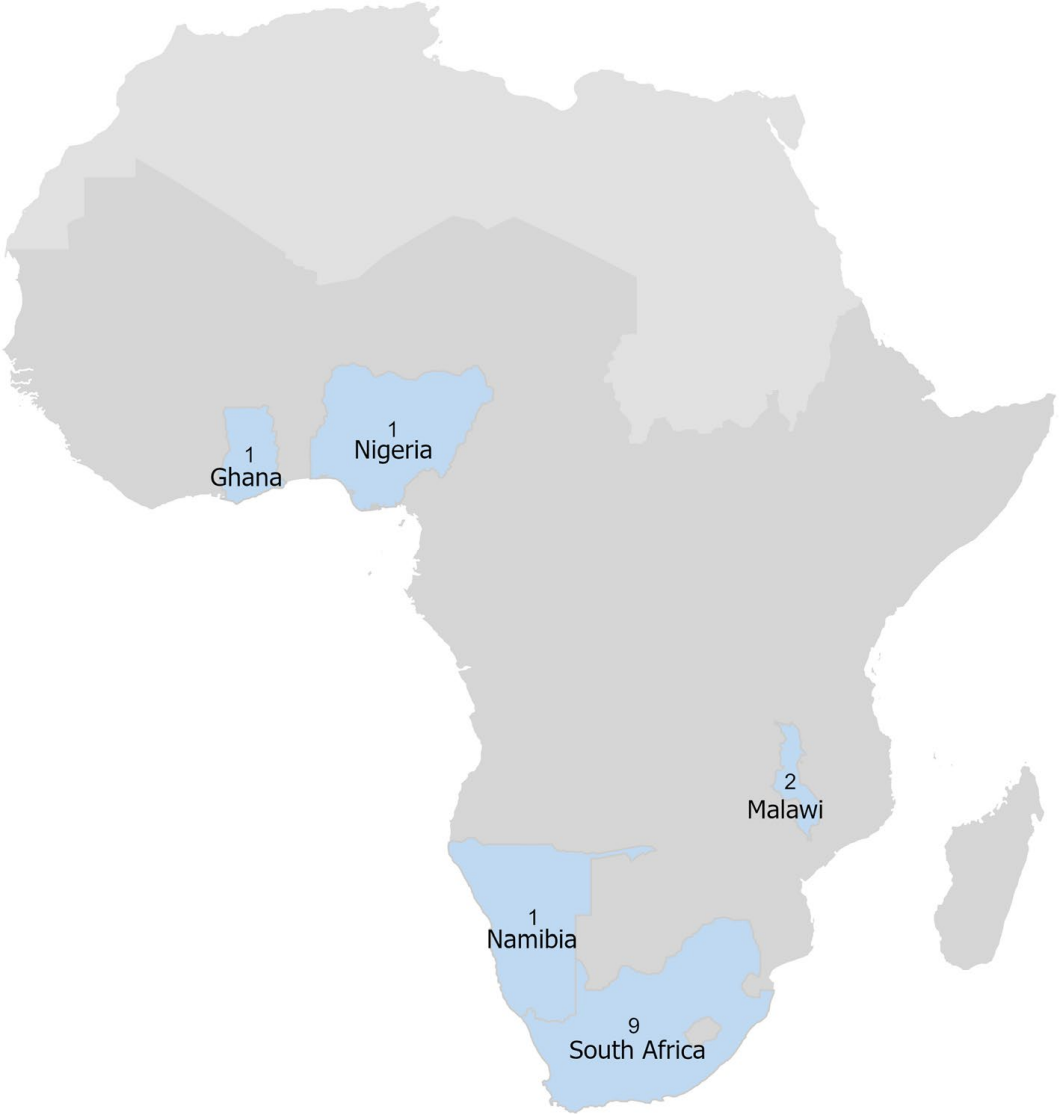
Number of papers by country among the 46 countries in Sub-Saharan Africa

The 14 papers comprised studies from just five countries, with nine of them being from South Africa alone [37–45]. Four of the studies from South Africa were conducted in the same area using similar methods by the same researchers [37, 40–42]. However, each of these focused on different aspects of air pollution and health including interactions between CD14 (-159) polymorphisms, air pollution and lung function [40], interaction of tumor necrosis factor alpha polymorphism and air pollution on lung function [41], short term fluctuations in ambient pollutants and respiratory health [42] and variations in pollutant exposure and respiratory health by context [37]. One study in South Africa covered respiratory health during the apartheid era and only included white children in the sample [45]. Ghana [46], Namibia [47], and Nigeria [48] each had one paper. Two studies on ambient air pollution exposures and non-communicable respiratory disease were from research conducted in Malawi [49, 50]. See Fig. 2 for a map of represented countries.

### Respiratory outcomes and study populations

Eight of the papers used measures of lung function as an outcome [39, 40, 42, 44, 46, 49–51]. The most common measure was of forced expiratory voumen (1 second) (FEV1), which was examined in each of these papers. Four of the papers examined associations of air pollutant exposures with forced vital capacity (FVC) [46, 49–51]. Two papers examined peak expiratory flow (PEF) and two papers assessed forced expiratory flow at 25 and 75% (FEF25-75) [39, 46]. Only one paper used fractional exhaled nitric oxide (FeNO) as an outcome of respiratory health [43].

Other papers, however, collected data on specific NCRDs, measured through diagnostic means. The papers’ ambient pollution and respiratory outcomes among schoolchildren in Durban, South Africa collected data on persistent asthma and chronic bronchitis and showed a prevalence of 14.8% and 4.1%, respectively [37]. In a study of South African children, the prevalence of asthma was 9.1% in the industrialized south and 5.0% in the north [39]. One South African study found that the prevalence of childhood asthma among a survey of 2,008 children was 5.0% [45]. In a study of South African schoolchildren, asthma was slightly more prevalent in the north, but disease severity was greater in the south [37]. A Nigerian study showed that the prevalence of respiratory symptoms and diseases of schoolchildren in Ibadan, Nigeria are as follows; 15% cough, 27% asthma, and 6% bronchitis. Two other studies from South Africa collected data on asthma and rhinitis.

Study populations varied among studies but were primarily schoolchildren. Other study populations included male e-waste workers [46], individuals exposed to a sulfur stockpile fire incident [38], and adult residents from specific regions [47, 49, 50] (Table 2).

**Table 2:** Papers that met the inclusion criteria with country where the study was performed, the study type, the time space of the research study and the quality score using the Appraisal Tool for Cross-Sectional Studies (AXIS) and the eight item Newcastle-Ottawa Scale for case/control studies

Citation	Country	Study Type	Study Span	Quality Score
Nti, et al., 2020	Ghana	Longitudinal/Cohort Study	Mar 2017- Nov 2018	8/9
Rylance, et al., 2020	Malawi	Cohort	Dec 2013 and Aug 2015	19/20
Nightingale, et al., 2019	Malawi	Cross-sectional	Aug 2014 - July 2015	19/20
Hamatui, et al., 2017	Namibia	Cross-Sectional	July 2015 - March 2016	14/20
Mustapha, et al., 2011	Nigeria	Cross-Sectional	Mar – Jun 2004	17/20
Naidoo, et al., 2013	South Africa	Cross-Sectional	Unspecified	18/20
Baatjies, et al., 2019	South Africa	Case-control	24 month period (dates unspecified)	7/8
Makamure, et al., 2016	South Africa	Cross-Sectional	2004-2005	15/20
Makamure, et al., 2017	South Africa	Cross-Sectional	2004-2005	16/20
Mentz, et al., 2018	South Africa	Cross-Sectional	Unspecified	16/20
Mentz, et al., 2019	South Africa	Cross-Sectional	Unspecified	16/20
Olaniyan, et al., 2020	South Africa	Longitudinal - Closed cohort	Baseline: Feb - Sept 2015 Follow up: Sept 2016	8/9
Reddy, et al., 2012	South Africa	Longitudinal cohort	2004-2005	8/9
Zwi, et al., 1990	South Africa	Prevalence Study / Cross Sectional	1980-1986	17/20

### Case definition

Case definition varied between articles. Most papers used questionnaires that recorded self-reported diagnoses [44–47, 49, 50, 52] or diagnosed respiratory condition through a standardized assessment tool using data on self-reported symptoms [37, 39–42, 51]. Only one paper relied on physician diagnoses [38]. The most common lung function measure was spirometry, a measure of the speed and volume of air movement through the lungs [37, 39, 40, 42, 44–46, 49–51]. None of the studies measured vital functions, such as heart rate or blood pressure. Three studies considered gene environment interactions [39, 40, 44]. None of the studies used biomarkers to diagnose chronic bronchitis or asthma.

### Study design

Longitudinal and repeated measurement studies: The study in Ghana conducted a longitudinal study of PM exposures and lung function, assessing e-waste workers and community controls at four time periods [46]. A study of PM exposure and lung function in schoolchildren in South Africa used a longitudinal design—testing children twice, one year apart [51]. One study from South Africa took lung and skin prick allergy tests for 1000 students cross-sectionally, but aggregated environmental measurements [37]. The studies from Durban, South Africa on lung function and air pollutant exposures used repeated measure designs—taking daily lung measurements over three weeks [39–41, 44]. A study in Malawi conducted a three-year longitudinal study on lung function and 48-hour PM and CO exposures, assessing subjects annually [50].

Cross-sectional studies: Many of the studies used cross-sectional designs. The study in Namibia measured both prevalence of disease and PM concentration for 107 residents of Windhoek over nine months [47]. The South African study of exposures to SO and asthma symptoms/lung function during a fire incident assessed associations based on duration and intensity of exposure retroactively, using exposure data collection at the time of the incident [38]. The study of traffic air pollution and respiratory illness cross-sectionally surveyed 1,397 schoolchildren ages 7-14 over the course of three months in 2004 [52]. One of the Malawian studies used a cross-sectional design while assessing a 48-hour exposure to pollutants [49]. The study of air pollution and health in Nigeria conducted a cross-sectional study with composite indices that used average CO and PM measurements taken on two occasions in schools [52].

Case-control studies: Only one study in our analysis used a case-control design. The study was conducted in South Africa among individuals who were residents at the time of a sulfur stockpile fire incident [38]. There were 76 cases, which constituted individuals who were residents of 18+ years, who did not have persistent lower respiratory symptoms (LRS) before the incident but developed symptoms one to six years after the incident; and 180 control individuals who were present but did not develop persistent LRS. Health outcomes were reported via questionnaires and confirmed by clinical examination [38]. Exposure to SO2 was measured using a sulfur dioxide exposure model and individual specific exposures based on local monitors during the time of the incident [38].

### Quality assessment

Only one of the evaluated studies was a case control and scored 7 on the Newcastle-Ottawa Quality Assessment Scale [38]. Four of the studies were classified as cohort studies and had scores ranging from 8 to 9 on the Newcastle-Ottawa Quality Assessment Scale [44, 46, 50, 51]. The majority of studies were cross-sectional, with nine studies classed as such [37, 39–42, 45, 47, 49, 52]. For these cross-sectional studies and using the AXIS assessment tool, we found that quality varied widely, with scores ranging from 14-20.

Across all study types, the most common shortcoming was failing to provide an adequate description of individuals who either did not respond to the survey, refused to participate, or were lost to follow up. Another common failing across study types was a lack of justification for the sample size selected for the study. In most studies, there was no indication that researchers calculated a sample size or a power calculation. In cohort studies, it was not always clear how comparable the exposed and unexposed cohorts were, complicating the interpretation of results. Even within the same urban area, it was not clear how comparable different areas were. Cross-sectional studies are considered to be subject to the most bias of the three study types—and given the nature of environmental exposures, the failure of most studies to adequately justify sample sizes and report on non-respondents is of concern.

### Exposure assessment

Various methods of assessing and measuring exposures were used in the reviewed studies. An early study in South Africa used air quality data from several government and private sources, along with climatological and wind pattern data, to account for factors dispersing and spreading pollution. These data were used to establish study and control areas for analysis of pollution exposure on FEV lung function in children [45]. Individually worn portable air samplers measured the breathing space of subjects in several of the studies. A study from Ghana used MetOne Aerocet 831 particle mass profiler portable air samplers incorporated into backpacks to collect PM2.5 and PM10 over 2- and 4-hour intervals [46]. Other studies sampled air within the vicinity of a school or other sampling locations. The study from Namibia [47] measured PM via particulate monitoring using the ASTM D1739 reference method [53, 54]. Monthly average PM level was calculated and allocated to individuals living within 1 km of the particular station. The research on a stockpile fire incident in South Africa measured SO2 concentrations using an Industrial Source Complex Short Term Model (ISCST3) to predict hourly 24-hour average concentrations through measurements taken shortly after the incident [38]. The paper on NO2 exposures in informal settlements in SA estimated average PM2.5 and NO2 concentrations at participants’ addresses through land-use regression models based on weekly measurements at 140 sites within the study area [51]. Estimated measures produced through the LUR were adjusted using spatial data layers. One study from Nigeria used CO and PM monitoring to create a composite index of traffic pollution [52]. CO measurements were conducted using handheld Gastec monitors, and PMx was sampled using a Dustmat portable environmental monitor. The studies from Durban, South Africa monitored gaseous air pollutant concentrations using nearly identical methods. Concentrations of NOx were measured using continuous gas-phase chemiluminescence; ultraviolet fluorescence spectrometry was used to measure SO2; and gravimetric sampling was used for PMxx [37, 39, 40, 42, 44, 55].

### Statistical and analytic methods

In most cases, statistical methods used standard methods of testing for difference or independence between groups and regression modelling. Researchers often conducted standard descriptive analyses to assess differences in sample demographics among exposure groups. These often included standard t-tests, tests for differences in medians, differences in proportions [49, 50], Spearman’s rank correlation coefficients [46, 56], and/or chi-square tests [38, 45] for independence between categorical variables [37, 46, 51]. Chi-square tests were also used for exploratory analyses to test difference between genotype frequency in diseased and non-diseased groups [40]

Models were created to attempt to control for various demographic confounders such as age, gender, and socioeconomic status. The cross-sectional study of NCRDs and air pollution in Malawi used multivariate regression including continuous measures for height and weight. This group used logistic regression to test for associations of pollutants with dichotomous clinical diagnoses, and linear regression modelling to test for associations with FEV measures [49]. Uniquely, this study also used Chi-square tests to assess possible selection bias in participants. The paper on e-waste workers controlled for various demographic factors and introduced terms to account for seasonality [46]. Regression models tested differences in lung function between measurement points for ambient exposures using multivariate regression, including confounders for age, gender, etc. in South Africa [51]. The paper on CD14 polymorphisms used a dominant coding model to assess genotypic effects in the models [40]. The paper on the stockpile incident used multiple logistic regression models, but did not specify which confounders were used [38]. The study of respiratory symptoms and particulate matter used bivariate and multivariate logistic regression with confounders [47].

Some studies used stepwise variable selection procedures to create multivariate models to test for associations between pollutant exposures and health outcomes, while controlling for other factors [45, 46, 50]. The cohort study from Malawi used an unspecified selection procedure with likelihood ratio tests under Maximum Likelihood Estimation (MLE) to select fixed-effect covariates for their mixed effects regression model [50]. In the study from children in Transvaal, SA claimed to use “stepwise logistic regression,” but it appears that the term is intended to mean that the researchers tested for bivariate associations [45].

Recent studies have tended to explore lag effects of exposures to health outcomes [37]. Among the papers we examined, only a few studies attempted any type of lag analysis. Some of the studies from Durban, South Africa modeled lag effects to account for acute and prior exposures to pollutants; they used generalized, linear models with random effects terms to account for within-subject effects [39, 40, 42, 44]. Two papers used generalized estimating equations (GEEs) to assess the pollution-symptoms associations and performed an explicit lag analysis of exposure and symptomatic outcomes, using single-exposure distributed lag models [41, 42]. The stockpile incident paper used a multivariate model controlling for confounders assessing subjects six years following exposure [38]. The cohort study of NCRD and air pollution in Malawi included a random intercept in their models to account for differences in individual baseline FEV function and to account for 24-hour clustering [50]. The study on e-waste workers was a longitudinal case-control study of changes in lung function given exposure to PM25 and PM10 and included a random slope [46].

One challenge in studies of pollutant exposures and health is that multiple pollutants often have common sources, which complicated the testing of hypotheses [57]. A common approach is to create composite indices of pollution-related risk factors to tease out the impact of correlated sets of factors with health [58, 59]. Out of the studies reviewed for this paper, only one used Principal Components Analysis (PCA) to create exposure indices [52]. The index was based on observed traffic counts, distance to road, and CO and PM concentrations. This Nigerian study used the composite indices in multivariate logistic regression models (with individual and household confounders) to test associations of symptomatic outcomes and self-reported indoor and outdoor exposures with the PCA indices [52].

Most studies did not attempt to account for missing data. Two performed multiple imputation for each pollutant and for each site, to account for missing hourly measures of airborne pollutants [41, 42]. A Markov Chain Monte Carlo method was used to determine hourly averages. One study used multiple imputation to account for missing exposure data from the monitors [44]. The article on CD14 polymorphisms and air pollutant exposures used multiple imputation methods to impute missing data for NOx monitors [40].

### Associations of risk factors with disease

Studies covered a wide range of exposures—including general air pollution (3 papers), PM2.5 (7 papers), PM10 (8 papers), ozone (1 paper), CO (6 papers), SO2 (8 papers), and NOx (7papers). Seven of the 14 papers selected for final analysis found significant results between an exposure and outcome of interest [41, 42, 45, 46, 49, 51]. The wide range of outcomes, exposures, and methods prevented us from providing a quantitative synthesis of the paper results. We opted to present a table of results summarizing all of the studies and the respective health indicators evaluated, along with the directionality (in terms of health) and significance of results (see Table 3).

**Table 3:** Results from the papers with specific outcomes and exposures. Direction of qualitative (good vs. poor) association of air pollutants and health indicators designated through up and down arrows. Significant results are denoted by a star and in bold. Dagger indicates association was shown in paper but the direction not reported

			Results						
First Author, year	Country	Health Indicators	Pollution/PM	PM2.5	PM10	CO	O3	NOx	SO2
Nti, 2020	Ghana	FEV1, FVC, FEV1/FVC, PEF, **FEF25-75**	-	*↓*	*↓*	-	-	-	-
Hamatui, 2017	Namibia	Episodes of phlegm and cough	*↑↓*	-	-	-	-	-	-
Naidoo, 2013	South Africa	Chronic Bronchitis, Persistent Asthma	-	*†*	*↓*	-	-	*†*	*↓*
Baatjies, 2013	South Africa	Total hours of exposure, cumulative exposure, peak exposure	-	-	-	-	-	-	*↑↓*
Olaniyan, 2020	South Africa	Rhinitis, Asthma, FEV1, FVC, FEV1/FVC, FEF25-75, Change in FEV1,**FENO**	-	*↑↓*	-	-	-	*↓* *	-
Zwi, 2020	South Africa	Rhinitis and **Asthma**	*↓* *	-	-	-	-	-	-
Reddy, 2012	South Africa	5 day average % change in intraday variability of FEV1	-	-	*↓*	-	-	*↓*	*↑*
Makamure, 2016	South Africa	5 day average % change in Intraday variability of FEV1, 5 day average of Intraday variability of PEF	-	-	*↓*	-	-	*↓*	*↓*
Makamure, 2017	South Africa	5 day average % change in Intraday variability of FEV1	-	-	*↓*	-	-	*↓*	*↓*
Mentz, 2018	South Africa	Respiratory Symptoms: (cough, wheeze, shortness of breath, chest tightness)	-	*†*	*↓* *	*†*	*†*	*↓* *	*↓* *
Mentz, 2019	South Africa	Change in **FEV1**	-	-	*†*	*†*	*†*	*↓* *	*†*
Mustapha, 2011	Nigeria	Asthma and Rhinitis	*↑*	*↑↓*	*↑*	*↑*	-	-	-
Nightingale, 2019	Malawi	Cough, Phlegm, Wheeze, Dyspnea, Functional Limitation, **Any symptom**, FEV1, FVC FEV1/FVC	-	*↓* *	-	*↑↓*	-	-	-
Rylance, 2020	Malawi	FEV1 and FVC	-	*†*	-	*†*	-	-	-

A study of male e-waste workers in Ghana found that percent change in FEF25-75 was significantly associated with exposure to PM10 when controlling for age, BMI, indoor cooking, seasonal variation, cigarette smoking, and job category [46]. A study on SO exposure during a sulfur stockpile fire incident found increased odds of having persistent lower respiratory symptoms/asthma at hour 15 controlling for age, gender, and smoking (OR 1.04 95%CI: 1.01-1.07) [38]. A study of South African schoolchildren (grade 4) tested for associations between asthma, asthma symptoms, and rhinitis and annual PM2.5 or annual NO2 concentrations, when controlling for age, gender, atopy, maternal smoking, dampness, visible mold growth, pets, cooking with paraffin, smoker in the home, and study area. Significant associations were found between ocular-nasal symptoms and PM2.5 (OR 1.49 95%CI: 1.08-2.06), ocular-nasal symptoms and NO2 (OR 1.85 95%CI: 1.19-2.86), wheezing and NO2 (OR 3.41 95%CI: 1.18-9.82), and asthma-symptom score >= 2 and NO2 (OR 1.70 95%CI: 1.04-2.78). All of the significant associations mentioned for this study lead to increased odds of asthma related symptoms. In addition, this study evaluated associations between lung function parameters and annual PM2.5 or annual NO2. The only significant association found was between FENO > 35ppb and NO2 (OR 3.09 95%CI 1.15-8.26) [51].

A study in South Africa found that respiratory symptoms occurred most frequently in children who went to schools polluted with smoke and SO2 [45]. A cross-sectional study in Malawi found that exposure to CO resulted in an increased odds of having any symptoms of respiratory diseases when controlling for weight, height, age, sex, and years of formal education (OR 1.46 95%CI 1.04-2.05) [49]. A study of schoolchildren in South Africa concluded that there is a significant positive association between exposure to PM2.5, SO2, NO2, and NO and increased respiratory symptoms when controlling for age, gender, race, school, caregiver smoking status, caregiver’s education, household income, season, asthma severity, and interactions between asthma severity and exposure [41]. Lastly, in another study of schoolchildren in South Africa, FEV1 decreased when NO and NO2 concentrations increased [42]. It should be noted that all statistically significant associations between exposures and outcomes had a directionality towards a worsened health status in the form of reduced lung function, increased presence of respiratory symptoms, or increased prevalence of chronic respiratory disease.

Many of the papers found null associations between exposures and outcomes of interest in their study populations. For example, a Namibian study found no significant associations between episodes of cough and phlegm and PM2.5 and PM10 [47]. One of the South African studies found no significant associations between asthma, chronic bronchitis, and symptoms of chronic respiratory conditions with PM10 and SO2 in schoolchildren, controlling for age, gender, race, education, smoker in the home, household income, and GTSM genotype [44]. Despite finding null associations in that study, there were significant regional differences in symptomatic profiles, suggesting industrialized areas have a higher risk for NCRDs than rural areas. See Table 3 for full results.

## Discussion

The paucity of research in SSA on associations of exposures to specific ambient air pollutants and NCRDs, such as chronic bronchitis, asthma, and allergic rhinitis, deserves special attention. Despite NCDs and NCRDs becoming of increasing concern in the region, only five countries were represented in this review. Nearly two thirds of papers were of research conducted in a single country, further highlighting the dire need for this kind of research in other areas. Countries such as Kenya, Ethiopia, and Uganda have large urban centers where air quality has been found to be poor [60, 61], but our results suggest that research on associations of air quality and health in these areas is desperately lacking. Given that it is estimated that air pollution was responsible for 1.1 million deaths—with ambient air pollution associated deaths increasing from 361,100 in 2015 to 383,000 in 2019—there is an urgent need for researchers both in and outside of SSA to work on understanding the links between air pollution and mortality in SSA populations [62].

In contrast to outdoor exposures to air pollutants, studies of respiratory disease that assessed associations with indoor exposures to pollutants (e.g biomass cooking fuels, smoking) are common throughout the literature. However, research in other regions has shown that chronic exposures to ambient air pollutants outside the home present specific risks to respiratory health [63, 64]. Emissions of air pollutants as a result of the use of fossil fuels is expected to increase two-fold in Africa by 2030 relative to 2012 [65]. Thus, research on the connections between ambient air pollution and NCRDs will be essential to generating knowledge that can inform efforts to reduce or respond to inevitable consequences to public health.

None of the studies included with this review examined socioeconomic impacts of airborne exposures with respiratory health. Evidence from other worldwide contexts suggests that poorer individuals and communities are exposed to higher concentrations of air pollutants [66–68]. Risks for respiratory diseases of all kinds are highest among the poorest groups of people around the world [69]. Research in sub-Saharan Africa has shown that health risks from indoor air pollution exposures vary along socioeconomic lines [28]. The papers we examined for this review indicated that there were significant relationships of ambient exposures with respiratory illness. Research should determine if these relationships differ between SES groups. As Africa urbanizes, fossil fuel and industrial emissions increase, and the socioeconomic gaps in air quality expand [70], the public health importance of air quality will become ever more salient [71].

Many studies evaluated lung function using spirometry. Only three studies examined gene-environment interactions [39, 40, 44]. Research might pivot away from traditional assessment and move toward recent developments in such fields as microbiome analysis. There is a dearth of studies looking at lung microbiota in SSA; such research could be of great importance to SSA contexts [72]. The field would benefit understanding of the associations between lung microbiota and exposure in SSA, especially given the recent findings that the cleaner biomass fuels showed no association with respiratory outcomes in adults [49, 50]. Future work could compare exposure responses between urban and rural populations; data from microbiome studies could help explain any differences between urban and rural populations. A challenge of studying broad environmental exposures is clearly delineating exposed and unexposed populations. The use of next generation bio-markers, such as miRNAs could help establish baseline responses in populations and aid in stratification by exposure status, potentially increasing the accuracy of traditional assessments such as spirometry [73–75].

Our review of the literature provides further insights into what may be driving the knowledge gaps around NCRD and air pollution in SSA. Though not restricted to studies of air pollution and health, low research budgets and high costs of placing and maintaining air pollution monitors prevent the collection of reliable data to assess exposure. Personal monitors are a way of averting the problem of deploying stationary monitors, but these also come with high investment in hardware and high costs for maintenance and research study management. Second, even when budgets exist to support the placement of monitors, they might miss crucial areas where exposure to air pollutants might be high. For example, we found that studies were often based on data collection in urban or highly developed areas. Monitors often seem to be placed in areas where pollution levels are known a priori to be high. While helpful for assessment of pollution levels in high-risk locations, the lack of comprehensive monitoring might complicate tests for associations between exposures and outcomes, given the lack of comparison areas. Further, not all areas where air pollution exposures might be high are considered to be traditionally urban. Densely populated rural areas, where bush burning and burning of trash is common, and vehicular fleets are older than those in urban areas, might lead to air pollution exposures comparable to traditionally urban areas. Studies from other areas of the world have suggested that air pollution exposures are also high in rural areas, and rural activities such as bush burning are a risk to health [76–78]. Finally, we found that the exposures, outcomes, study designs, and methodologies used in the studies varied widely; so much so, that we were unable to provide a quantitative synthesis of the results. Researchers might attempt to replicate the methods of one or more of the studies in their own regions, in order to be able to make comparisons between contexts, and provide a source for Continent wide estimates.

We call for a concerted effort to develop national or regional air quality monitoring systems, across urban and rural contexts, that can provide publicly available and cost-free data in real time. The development of new methods of ambient pollution measurement tailored to low resource settings, such as passive samplers to measure light-absorbing carbon pollutants, are also critical for generation of data on air pollution in SSA [79]. In addition, the use of novel study designs, such as the assignment of PM2.5 exposure levels using modest sensor networks and structured questionnaires to assess activity levels as was done in Ghana [80], can be used to develop methodologies tailored to the SSA context. Overall, work on advancing any type of assessment technology for SSA would be a major contribution to expanding the ability to test hypotheses of air pollution impacts on health.

Our review has indicated that the capacity for conducting studies on air pollution and health exists. This capacity and operational knowledge should be deployed more widely throughout the continent. As such, air pollution and health should be a priority for public health research efforts at all levels. International funders and local governments should increase funding for data collection and to hire professionals within country to manage and maintain such efforts. Given the potentially serious impacts of pollution on health, researchers should place air pollution studies to the forefront of their work.

This literature review has some limitations. First, limiting the study to only the three major search databases might have resulted in missing some important research. Some studies of air pollution and NCRDs might have appeared in small or regional journals not listed in one of the three databases. Other studies might be encapsulated in government or country-level reports that will be missed in a review using these methods. Our study, however, sought to characterize the state of research as available to investigators worldwide, and as a result we felt that restricting our search to these databases was appropriate. Another limitation is in the non-specific nature of assessing NCRDs based on self-report, diagnoses provided by a physician, or through an assessment tool. A more expansive review or meta-analysis that searched for symptomatic profiles that might indicate NCRDs might provide additional insights.

Climate change might also impact the effects of air pollution on NCRDs in SSA. Model projections show a decrease in PM2.5 particles near the equator because of increased precipitation, while in southern Africa a projected decrease in precipitation will likely result in an increase in atmospheric PM2.5 [81]. The complexity of potential outcomes will be further compounded by the impact of urbanization. For example, in West Africa, a region characterized by a sensitive monsoon system and precipitation variability, explosive urbanization could result in regional climatic differences from the interactions between aerosols and cloud cover, and changes in atmospheric chemistry [82]. Climate change adaptation will require that research work to develop a clearer understanding of the impact of air pollution on NCRDs in SSA populations.

The lack of research drawing explicit relationships of outdoor air pollution and NCRDs is striking. We believe that we have demonstrated a need for new and innovative research to fill this knowledge gap. Research should also inform and support targeted intervention to improve air quality and protect public health. Further, research in SSA could expand to include other types of health conditions known to be associated with, or which can be worsened by exposures to air pollutants. It is our hope that this review acts as a “call to arms” to all researchers working in the region on air quality, respiratory health, and environmental health. However, we also ask that international funding sources support and build capacity for in-country expertise to help bolster awareness and provide data that can answer relevant important and novel questions.

## Data Availability

Not applicable.

## Abbreviations

NCD: Non-communicable disease
NCRD: Non-communicable respiratory disease
SSA: Sub-Saharan Africa
AR: Allergic rhino-conjunctivitis
ARI: Acute respiratory infection
PM: Particulate matter
SO: Sulfuric oxide
NO: Nitrous oxide
O3: Ozone
WHO: World Health Organization

## References

[1] United Nations Development Program. About Sub-Saharan Africa: Africa at a turning point: United Nations Development Program; 2021. Available from: https://www.africa.undp.org/content/rba/en/home/regioninfo.html. Accessed 18 Aug 2021.

[2] World Bank. World Bank Open Data: World Bank Open Data; 2020. Available from: https://data.worldbank.org/indicator/SP.URB.TOTL.IN.ZS?locations=ZG. Accessed 18 Aug 2021.

[3] Hoornweg D, Pope K (2017). Population predictions for the world’s largest cities in the 21st century. Environ Urban.

[4] Pauleit S, Coly A, Fohlmeister S, Gasparini P, Jørgensen G, Kabisch S, et al.Urban vulnerability and climate change in Africa: a multidisciplinary approach. vol. 4 of Future city. Switzerland: Springer International Publishing AG; 2015.

[5] World Bank. Life expectancy at birth, total (years): World Bank Open Data; 2020. Available from: https://data.worldbank.org/indicator/SP.DYN.LE00.IN?locations=ZG. Accessed 18 Aug 2021.

[6] Gouda HN, Charlson F, Sorsdahl K, Ahmadzada S, Ferrari AJ, Erskine H, et al.Burden of non-communicable diseases in sub-Saharan Africa, 1990–2017: results from the Global Burden of Disease Study 2017. Lancet Glob Health. 2019;7(10):e1375–87. 10.1016/S2214-109X(19)30374-2 31537368

[7] Seraphin TP, Joko-Fru WY, Kamaté B, Chokunonga E, Wabinga H, Somdyala NIM (2021). Rising prostate cancer incidence in Sub-Saharan Africa: a trend analysis of data from the African Cancer Registry Network. Cancer Epidemiol Biomark Prev.

[8] Joko-Fru WY, Jedy-Agba E, Korir A, Ogunbiyi O, Dzamalala CP, Chokunonga E (2020). The evolving epidemic of breast cancer in sub-Saharan Africa: Results from the African Cancer Registry Network. Int J Cancer.

[9] Twagirumukiza M, De Bacquer D, Kips JG, de Backer G, Stichele RV, Van Bortel LM (2011). Current and projected prevalence of arterial hypertension in sub-Saharan Africa by sex, age and habitat: an estimate from population studies. J Hypertens.

[10] Mohsen IbrahimPM, Albertino DamascenoP (2012). Hypertension in developing countries. Lancet (Br Ed).

[11] Motala AA (2002). Diabetes trends in Africa. Diabetes Metab Res Rev.

[12] Kengne AP, Bentham J, Zhou B, Peer N, Matsha TE, Bixby H (2017). Trends in obesity and diabetes across Africa from 1980 to 2014: an analysis of pooled population-based studies. Int J Epidemiol.

[13] Schneider M, Bradshaw D, Steyn K, Norman R, Laubscher R. (2009). Poverty and non-communicable diseases in South Africa. Scand J Public Health.

[14] De-Graft Aikins A, Unwin N, Agyemang C, Allotey P, Campbell C, Arhinful D (2010). Tackling Africa’s chronic disease burden: from the local to the global. Glob Health.

[15] Tesema AG, Ajisegiri WS, Abimbola S, Balane C, Kengne AP, Shiferaw F (2020). How well are non-communicable disease services being integrated into primary health care in Africa: A review of progress against World Health Organization’s African regional targets. PLoS ONE.

[16] Atun R, Jaffar S, Nishtar S, Knaul FM, Barreto ML, Nyirenda M (2013). Improving responsiveness of health systems to non-communicable diseases. Lancet.

[17] Wang Q, Brenner S, Kalmus O, Banda HT, De Allegri M. The economic burden of chronic non-communicable diseases in rural Malawi: an observational study. BMC Health Serv Res. 2016;16(1). Available from: https://dx.doi.org/10.1186/s12913-016-1716-8. 10.1186/s12913-016-1716-8 PMC5007731 27582052

[18] Environmental Protection Agency. Particulate Matter, Air and Radiation, US EPA: Environmental Protection Agency; 2011. Available from: http://www.epa.gov/pm/. Accessed 18 Aug 2021.

[19] Anderson JO, Thundiyil JG, Stolbach A (2012). Clearing the air: a review of the effects of particulate matter air pollution on human health. J Med Toxicol.

[20] Liu L, Yu LY, Mu HJ, Xing LY, Li YX, Pan GW (2014). Shape of concentration-response curves between long-term particulate matter exposure and morbidities of chronic bronchitis: a review of epidemiological evidence. J Thorac Dis.

[21] Dimitrova R, Lurponglukana N, Fernando HJS, Runger GC, Hyde P, Hedquist BC (2012). Relationship between particulate matter and childhood asthma – basis of a future warning system for central Phoenix. Atmos Chem Phys.

[22] Keeler GJ, Dvonch T, Yip FY, Parker EA, Isreal BA, Marsik FJ (2002). Assessment of personal and community-level exposures to particulate matter among children with asthma in Detroit, Michigan, as part of Community Action Against Asthma (CAAA). Environ Health Perspect.

[23] Lin M, Chen Y, Burnett RT, Villeneuve PJ, Krewski D (2002). The influence of ambient coarse particulate matter on asthma hospitalization in children: case-crossover and time-series analyses. Environ Health Perspect.

[24] Lin HW, HuangFu H, Li YJ, Han R (2019). Particulate matter pollution and allergic rhinitis. Lin Chuang Er Bi Yan Hou Ke Za Zhi.

[25] Gillespie-Bennett J, Pierse N, Wickens K, Crane J, Howden-Chapman P (2011). The respiratory health effects of nitrogen dioxide in children with asthma. Eur Respir J.

[26] Burte E, Leynaert B, Marcon A, Bousquet J, Benmerad M, Bono R (2020). Long-term air pollution exposure is associated with increased severity of rhinitis in two European cohorts. J Allergy Clin Immunol.

[27] Agbo KE, Walgraeve C, Eze JI, Ugwoke PE, Ukoha PO, Van Langenhove H (2021). A review on ambient and indoor air pollution status in Africa. Atmos Pollut Res.

[28] Emmelin A, Wall S (2007). Indoor air pollution: a poverty-related cause of mortality among the children of the world. Chest.

[29] Kurmi OP, Lam KBH, Ayres JG (2012). Indoor air pollution and the lung in low- and medium-income countries. Eur Respir J.

[30] Masekela R (2020). Vanker A. Lung Health in Children in Sub-Saharan Africa: Addressing the Need for Cleaner Air. Int J Environ Res Public Health.

[31] Jafta N, Jeena PM, Barregard L, Naidoo RN. Association of childhood pulmonary tuberculosis with exposure to indoor air pollution: a case control study. BMC Public Health. 2019;19(1). Available from: https://dx.doi.org/10.1186/s12889-019-6604-9. 10.1186/s12889-019-6604-9 PMC6407209 30845944

[32] Pathak U, Gupta NC, Suri JC (2020). Risk of COPD due to indoor air pollution from biomass cooking fuel: a systematic review and meta-analysis. Int J Environ Health Res.

[33] Thacher JD, Emmelin A, Madaki AJK, Thacher TD (2013). Biomass fuel use and the risk of asthma in Nigerian children. Respir Med.

[34] Coker E, Kizito S. (2018). A narrative review on the human health effects of ambient air pollution in Sub-Saharan Africa: an urgent need for health effects studies. Int J Environ Res Public Health.

[35] Downes MJ, Brennan ML, Williams HC, Dean RS (2016). Development of a critical appraisal tool to assess the quality of cross-sectional studies (AXIS). BMJ Open.

[36] Wells G, Shea B, O’Connell D, Peterson J, Welch V, Losos M, et al. The Newcastle-Ottawa Scale (NOS) for assessing the quality of nonrandomised studies in meta-analyses. Ottawa: The Ottawa Hospital; 2014.

[37] Naidoo RN, Robins TG, Batterman S, Mentz G, Jack C (2013). Ambient pollution and respiratory outcomes among schoolchildren in Durban, South Africa [Journal Article]. Sajch.

[38] Baatjies R, Adams S, Cairncross E, Omar F, Jeebhay MF. Factors associated with persistent lower respiratory symptoms or asthma among residents exposed to a sulphur stockpile fire incident [Journal Article]. 2019;16(3). 10.3390/ijerph16030438 PMC6388145 30717374

[39] Makamure MT, Reddy P, Chuturgoon A, Naidoo RN, Mentz G, Batterman S (2016). Tumour necrosis factor alpha polymorphism (TNF-308alpha G/A) in association with asthma related phenotypes and air pollutants among children in KwaZulu-Natal [Journal Article]. Asian Pac J Allergy Immunol.

[40] Makamure MT, Reddy P, Chuturgoon A, Naidoo RN, Mentz G, Batterman S (2017). Interaction between ambient pollutant exposure, CD14 (-159) polymorphism and respiratory outcomes among children in Kwazulu-Natal, Durban [Journal Article]. Hum Exp Toxicol.

[41] Mentz G, Robins TG, Batterman S, Naidoo RN (2018). Acute respiratory symptoms associated with short term fluctuations in ambient pollutants among schoolchildren in Durban, South Africa [Journal Article]. Environ Pollut.

[42] Mentz G, Robins TG, Batterman S, Naidoo RN (2019). Effect modifiers of lung function and daily air pollutant variability in a panel of schoolchildren [Journal Article]. Thorax.

[43] Olaniyan T, Dalvie MA, Röösli M, Naidoo R, Künzli N, de Hoogh K (2019). Asthma-related outcomes associated with indoor air pollutants among schoolchildren from four informal settlements in two municipalities in the Western Cape Province of South Africa. Indoor air.

[44] Reddy P, Naidoo RN, Robins TG, Mentz G, Li H, London SJ (2012). GSTM1 and GSTP1 gene variants and the effect of air pollutants on lung function measures in South African children [Journal Article]. Am J Ind Med.

[45] Zwi S, Davies JC, Becklake MR, Goldman HI, Reinach SG, Kallenbach JM (1990). Respiratory health status of children in the eastern Transvaal highveld [Journal Article]. S Afr Med J.

[46] Amoabeng Nti AA, Arko-Mensah J, Botwe PK, Dwomoh D, Kwarteng L, Takyi SA (2020). Effect of particulate matter exposure on respiratory health of e-waste workers at Agbogbloshie, Accra, Ghana. Int J Environ Res Public Health..

[47] Hamatui N, Beynon C (2017). Particulate matter and respiratory symptoms among adults living in Windhoek, Namibia: a cross sectional descriptive study. Int J Environ Res Public Health..

[48] Mbatchou Ngahane BH, Afane Ze E, Chebu C, Mapoure NY, Temfack E, Nganda M (2015). Effects of cooking fuel smoke on respiratory symptoms and lung function in semi-rural women in Cameroon [Journal Article]. Int J Occup Environ Health.

[49] Nightingale R, Lesosky M, Flitz G, Rylance SJ, Meghji J, Burney P (2019). Noncommunicable respiratory disease and air pollution exposure in Malawi (CAPS): a cross-sectional study. Am J Respir Crit Care Med.

[50] Rylance S, Jewell C, Naunje A, Mbalume F, Chetwood JD, Nightingale R (2020). Non-communicable respiratory disease and air pollution exposure in Malawi: a prospective cohort study. Thorax.

[51] Olaniyan T, Jeebhay M, Röösli M, Naidoo RN, Künzli N, de Hoogh K (2020). The association between ambient NO(2) and PM(2.5) with the respiratory health of school children residing in informal settlements: A prospective cohort study [Journal Article]. Environ Res.

[52] Mustapha BA, Blangiardo M, Briggs DJ, Hansell AL (2011). Traffic air pollution and other risk factors for respiratory illness in schoolchildren in the niger-delta region of Nigeria. Environ Health Perspect.

[53] Kwata MG. Comparison of methods for measurement of dust deposition in South African mining sectors. Pretoria: Dissertation, University of Pretoria; 2014.

[54] Malakootian M, Ghiasseddin M, Akbari H, Jaafarzadeh-Haghighi Fard NA (2013). Urban dust fall concentration and its properties in Kerman City, Iran. Health Scope.

[55] Mehanna N, Mohamed N, Wordofa M, Abera D, Mesfin A, Wolde M (2018). Allergy-related disorders (ARDs) among Ethiopian primary school-aged children: Prevalence and associated risk factors [Journal Article]. PLoS ONE.

[56] Ayuk AC, Eze JN, Edelu BO, Oguonu T. (2018). The prevalence of allergic diseases among children with asthma: What is the impact on asthma control in South East Nigeria? [Journal Article]. Niger J Clin Pract.

[57] Analitis A, De’ Donato F, Scortichini M, Lanki T, Basagana X, Ballester F (2018). Synergistic Effects of Ambient Temperature and Air Pollution on Health in Europe: Results from the PHASE Project. Int J Environ Res Public Health.

[58] Occelli F, Lanier C, Cuny D, Deram A, Dumont J, Amouyel P (2020). Exposure to multiple air pollutants and the incidence of coronary heart disease: A fine-scale geographic analysis. Sci Total Environ.

[59] Cao R, Wang Y, Huang J, Zeng Q, Pan X, Li G (2021). The construction of the air quality health index (AQHI) and a validity comparison based on three different methods. Environ Res.

[60] Desouza P (2020). Air pollution in Kenya: a review. Air Quality. Atmos Health.

[61] Singh A, Ng’ang’a D, Gatari MJ, Kidane AW, Alemu ZA, Derrick N, et al.Air quality assessment in three East African cities using calibrated low-cost sensors with a focus on road-based hotspots. Environ Res Commun. 2021; 3(7). Copyright - Ⓒcopy; 2021. This work is published under http://creativecommons.org/licenses/by/4.0/ (the “License”). Notwithstanding the ProQuest Terms and Conditions, you may use this content in accordance with the terms of the License; Last updated - 2021-07-27; SubjectsTermNotLitGenreText - Addis Ababa Ethiopia; Ethiopia; Kampala Uganda; Uganda. Accessed 18 Aug 2021.

[62] Fisher S, Bellinger DC, Cropper ML, Kumar P, Binagwaho A, Koudenoukpo JB (2021). Air pollution and development in Africa: impacts on health, the economy, and human capital. Lancet Planet Health.

[63] Sweileh WM, Al-Jabi SW, Zyoud SH, Sawalha AF. Outdoor air pollution and respiratory health: a bibliometric analysis of publications in peer-reviewed journals (1900–2017). Multidiscip Respir Med. 2018;13(1). Available from: https://dx.doi.org/10.1186/s40248-018-0128-5. 10.1186/s40248-018-0128-5 PMC5984296 29881545

[64] Kurt OK, Zhang J, Pinkerton KE (2016). Pulmonary health effects of air pollution. Curr Opin Pulm Med.

[65] Marais EA, Silvern RF, Vodonos A, Dupin E, Bockarie AS, Mickley LJ (2019). Air Quality and Health Impact of Future Fossil Fuel Use for Electricity Generation and Transport in Africa. Environ Sci Technol.

[66] Kravitz-Wirtz N, Teixeira S, Hajat A, Woo B, Crowder K, Takeuchi D (2018). Early-life air pollution exposure, neighborhood poverty, and childhood asthma in the United States, 1990–2014. Int J Environ Res Public Health.

[67] Lipfert FW (2004). Air pollution and poverty: does the sword cut both ways?. J Epidemiol Community Health (1979-).

[68] Hajat A, Hsia C, O’Neill MS (2015). Socioeconomic Disparities and Air Pollution Exposure: a Global Review. Curr Environ Health Rep.

[69] Schraufnagel DE, Blasi F, Kraft M, Gaga M, Finn PW, Rabe KF (2013). An official American Thoracic Society/European Respiratory Society policy statement: disparities in respiratory health. Am J Respir Crit Care Med.

[70] Rooney MS, Arku RE, Dionisio KL, Paciorek C, Friedman AB, Carmichael H (2012). Spatial and temporal patterns of particulate matter sources and pollution in four communities in Accra, Ghana. Sci Total Environ.

[71] Abera A, Friberg J, Isaxon C, Jerrett M, Malmqvist E, Sjostrom C (2021). Air Quality in Africa: Public Health Implications. Annu Rev Public Health..

[72] Huffnagle G, Dickson R, Lukacs N. (2017). The respiratory tract microbiome and lung inflammation: a two-way street. Mucosal Immunulogy.

[73] Heffler E, Allegra A, Pioggia G, Picardi G, Musolino C, Gangemi S. (2017). MicroRNA profiling in asthma: potential biomarkers and therapeutic targets. Am J Respir Cell Mol Biol.

[74] Kho AT, McGeachie MJ, Moore KG, Sylvia JM, Weiss ST, Tantisira KG (2018). Circulating microRNAs and prediction of asthma exacerbation in childhood asthma. Respir Res..

[75] Rupani H, Sanchez-Elsner T, Howarth P. (2012). MicroRNAs and respiratory diseases. Eur Respir J.

[76] Wang Y, Puthussery JV, Yu H, Liu Y, Salana S, Verma V. (2022). Sources of cellular oxidative potential of water-soluble fine ambient particulate matter in the Midwestern United States. J Hazard Mater.

[77] Akpinar-Elci M, Coomansingh K, Blando J, Mark L (2015). Household bush burning practice and related respiratory symptoms in Grenada, the Caribbean. J Air Waste Manag Assoc.

[78] Hickman JE, Andela N, Tsigaridis K, Galy-Lacaux C, Ossohou M, Bauer SE. Reductions in NO2 burden over north equatorial Africa from decline in biomass burning in spite of growing fossil fuel use, 2005 to 2017. Proc Natl Acad Sci. 2021;118(7). Available from: https://www.pnas.org/content/118/7/e2002579118. Accessed 18 Aug 2021. 10.1073/pnas.2002579118 PMC7896302 33558224

[79] Clark LP, Sreekanth V, Bekbulat B, Baum M, Yang S, Baylon P (2020). Developing a low-cost passive method for long-term average levels of light-absorbing carbon air pollution in polluted indoor environments. Sensors.

[80] Amegah AK, Dakuu G, Mudu P, Jaakkola J (2022). Particulate matter pollution at traffic hotspots of Accra, Ghana: levels, exposure experiences of street traders, and associated respiratory and cardiovascular symptoms. J Expo Sci Environ Epidemiol..

[81] Silva RA, Lamarque JF, SD T, William CJ, Faluvegi G, Folberth GA (2017). Future global mortality from changes in air pollution attributable to climate change. Nat Clim Chang.

[82] Knippertz P, Evans MJ, Field PR, Fink A, Liousse C, Marsham JH (2015). The possible role of local air pollution in climate change in West Africa. Nat Clim Chang.

